# An Antisense *yycF* RNA Modulates Biofilm Organization of Methicillin-Resistant *Staphylococcus aureus* and Pathogenicity in a Rat Model of Osteomyelitis

**DOI:** 10.3390/antibiotics10050603

**Published:** 2021-05-19

**Authors:** Shizhou Wu, Yunjie Liu, Lei Lei, Hui Zhang

**Affiliations:** 1 Department of Orthopedics, West China Hospital, Sichuan University, Chengdu 610041, China; wushizhou1990@wchscu.cn; 2 Department of Nutrition, Food Hygiene and Toxicology, West China School of Public Health, Sichuan University, Chengdu 610041, China; liuyunjie@scu.edu.cn; 3 Department of Preventive Dentistry, West China Hospital of Stomatology, Sichuan University, Chengdu 610041, China

**Keywords:** antisense, biofilm formation, *Staphylococcus aureus*, YycFG, osteomyelitis

## Abstract

*Staphylococcus aureus* (*S. aureus*) is one of most common opportunistic pathogens and is attributed to several human infections. The increasing incidence of methicillin-resistant *S. aureus* (MRSA) is a serious clinical threat for osteomyelitis crisis. The YycFG two-component system of *S. aureus* regulates genes associated with biofilm formation. To investigate the potential role of an antisense *yycF* RNA in the regulation of transcription levels of *yycF* and associated effects on biofilm formation and pathogenicity, antisense *yycF* (AS*yycF*) RNA was detected by RT-PCR and 5′ RACE assays. AS*yycF* overexpression mutants were constructed, and the biofilm biomass was determined by crystal violet microtiter assay and scanning electron microscopy (SEM). Quantitative RT-PCR and Western blotting analyses were used to detect whether AS*yycF* overexpression inhibited the transcription and translation of biofilm-related genes. Then, a rat tibial infective model was used to evaluate the pathogenicity of AS*yycF* overexpression in vivo. AS*yycF* transcription led to reductions in YycF production and biofilm formation. Overexpression of AS*yycF* inhibited the transcription and translation of biofilm-related genes. The sensitivity to vancomycin was improved in AS*yycF*-overexpressing MRSA. Furthermore, AS*yycF* inhibited MRSA invasion in a rat tibial infection model. From this study, the expression of the YycF protein was found to be inversely correlated with different levels of AS*yycF* transcription. The biofilm biomass and pathogenicity decreased in the AS*yycF*-overexpressing mutant. Thus, the current evidence may support AS*yycF* as a supplementary strategy for managing *S. aureus* and MRSA infections.

## 1. Introduction

*Staphylococcus aureus* is one of most common opportunistic pathogens and is attributed to several human and animal infections [1,2]. As a main causative agent of osteomyelitis (OM) worldwide, *S. aureus* accounts for 50% to 80% of OM cases [3]. Antibiotics such as penicillin, methicillin, and glycopeptides have been used to fight *S. aureus* infections for several decades. However, most antibiotics used to treat *S. aureus* infections not only have several side effects but also induce *S. aureus* to gain drug resistance against most antibiotics [4]. Although there is a debate on whether the abuse of antibiotics catalyzes the emergence of methicillin resistance in *S. aureus* (MRSA), the number of clinical isolates with MRSA is increasing, constituting a serious problem [5]. There is a robust demand to develop highly effective antibacterial agents to defeat this threatening resistance crisis.

*S. aureus* acts as a biofilm producer and is an essential factor in its pathogenicity [6]. Biofilm formation is a physical barrier that allows bacteria to evade the immune system and enhances resistance to conventional antibiotics up to 1000-fold, which results in therapeutic failures. Drug resistance in biofilms is mostly associated with the slow growth of bacteria and the difficult diffusion of antibacterial agents. Therefore, tolerance will be lost in planktonic form with the dispersion of biofilms [7,8]. In biofilm organization, polysaccharide intercellular adhesin (PIA) is the major extracellular polysaccharide (EPS) encoded by the *ica* gene that participates in pathogenesis, including biofilm formation and immune evasion [9,10].

Two-component regulatory systems (TCSs) exist ubiquitously in bacteria to adapt to stimuli and nutrition alterations from the external environment [11,12]. Typically, the TCS contains a histidine kinase (HK), which can autophosphorylate a conserved histidine residue in response to extracellular stimulus, and a response regulator (RR), which can transfer the phosphoryl group from HK [13]. Among 16 TCSs in *S. aureus*, only YycFG is essential for bacterial viability [14]. In our previous study, YycFG modulated *ica* genes that are involved in biofilm development, and the expression of YycFG was significant in MRSA strains [6]. From this point of view, YycFG has the potential to be a target for the treatment of MRSA infections.

Antisense RNA (AS RNA), a kind of noncoding RNA, can be bound by base-pairing to the target messenger RNA (mRNA). Their interactions result in the formation of an RNA duplex structure, which generally regulates gene expression and downstream functions [15,16]. In our previous studies, endogenous *vicR* antisense and *walR* antisense RNAs were identified in *Streptococcus mutans* and *Enterococcus faecalis*, respectively [15,16]. According to these investigations, the effect of antisense RNA has an inverse association with TCS expression and biofilm formation [10]. In the present study, whether a potential antisense *yycF* RNA exists was hypothesized, and whether the potential AS*yycF* is specifically associated with the regulation of YycF function in *S. aureus* and MRSA strains was also investigated.

## 2. Results

### 2.1. ASyycF Modulated Bacterial Growth and Biofilm Organization

**Figure 1:**
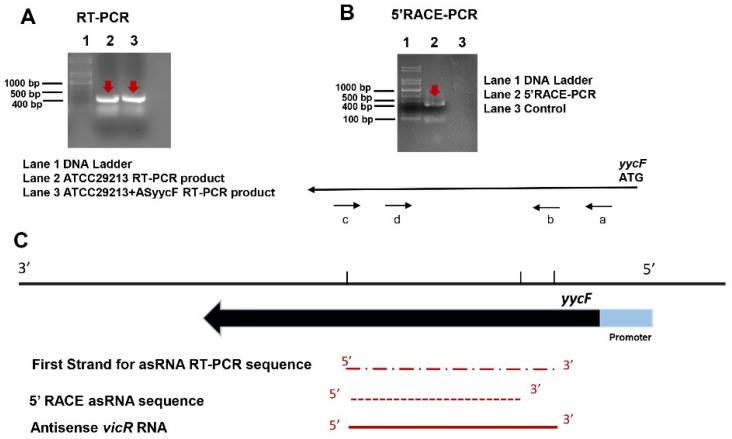
Detection of antisense *yycF* RNA. (**A**) Total RNA samples were isolated from *S. aureus* to detect AS*yycF* RNA transcription by first cDNA strand synthesis and RT-PCR (red arrows). (**B**) Detection of the 5′ terminus of the AS*yycF* transcription by 5′ RACE (red arrows): (a) gene-specific outer primer; (b) gene-specific inner primer; (c) and (d) are 5′ RACE outer and inner primers, respectively. (**C**) Schematic of AS*yycF* showing that transcription starts within the 5′ terminus in the *yycF* open reading frame (ORF). The full length for AS*yycF* RNA is approximately 400 bp.

**Figure 2:**
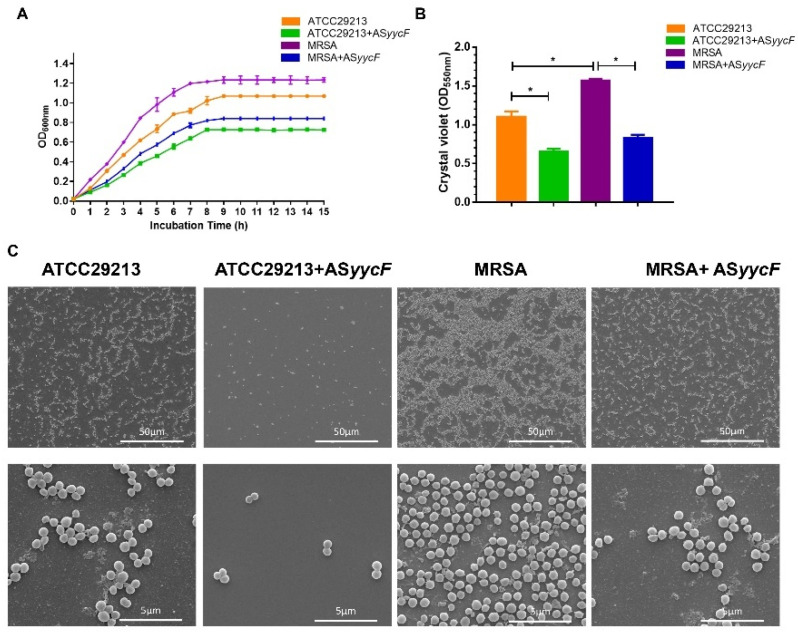
AS*yycF* modulated the bacterial growth and biofilm organization. (**A**) The growth curves for the *Staphylococcus aureus*. (**B**) Biomass was quantified by crystal violet staining. Optical densities at 600 nm were measured (*n* = 10, * *p* < 0.05). (**C**) SEM images of *S. aureus* ATCC29213, and methicillin-resistant *Staphylococcus aureus* (MRSA) strains after AS*yycF* overexpression.

We investigated whether a potential antisense RNA was specifically associated with *yycF* using first-strand cDNA synthesis. Total RNA was prepared from the ATCC29213 strain grown as planktonic or biofilm cultures in TSB medium. These RNA samples were used as templates for first-strand DNA synthesis using *yycF* antisense-specific (SAPCR) and sense-specific primers (SAAS, Table 1). AS*yycF* transcript of the predicted size (approximately 400 bases) was detected, as shown in Figure 1A. We used 5′ RACE for additional confirmation of AS*yycF*, and the primers used in the PCRs are listed in Table 1. The position of primers used for 5′ RACE assays and gel electrophoresis of the 5′ RACE PCR amplicon are indicated in Figure 1B. The sequence predicts that the 5′ terminus of AS *yycF* begins within the *yycF* coding sequences. The growth curves of ATCC29213, AS*yycF* ATCC29213, MRSA, and AS*yycF* MRSA strains were compared in three independent experiments. For the growth curve, AS*yycF*-overexpressing MRSA and ATCC29213 strains were delayed from entering into log phase by three hours compared with the MRSA and ATCC29213 strains (Figure 2A). By quantitative crystal violet microtiter assays, the biomasses of AS*yycF*-overexpressing MRSA and ATCC29213 biofilms showed nearly twofold decreases compared with MRSA and ATCC29213 biofilms (Figure 2B). SEM observations revealed that MRSA and ATCC29213 strains highly accumulated extracellular matrix compared with AS*yycF*-overexpressing MRSA and ATCC29213 strains (Figure 2C).

### 2.2. Effect of Biofilm Formation on Antibiotic Sensitivity

**Figure 3:**
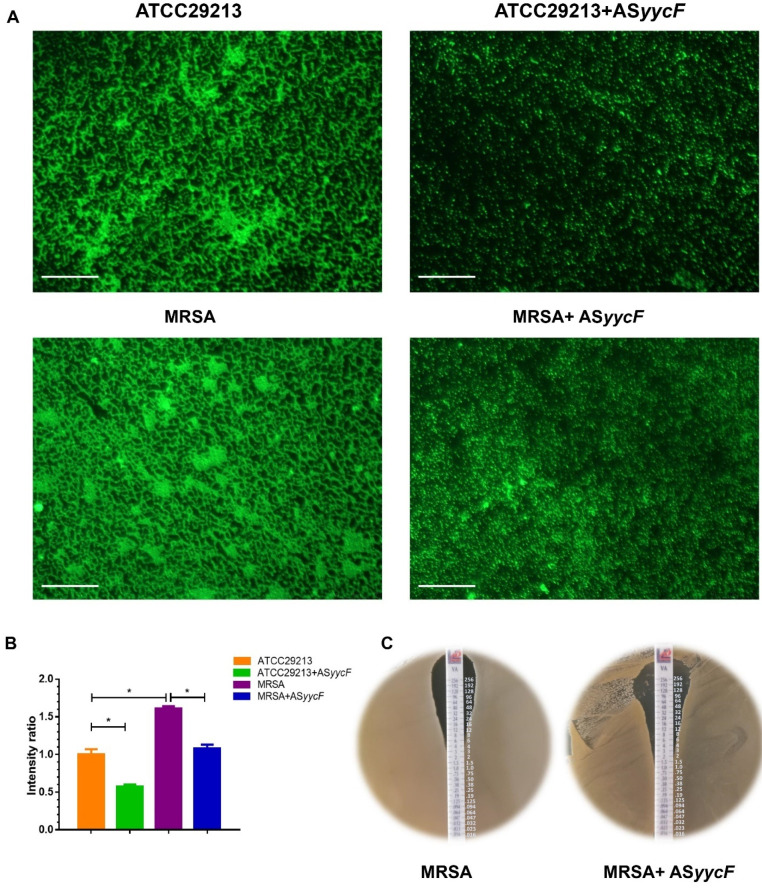
Effect of biofilm formation on antibiotics sensitivity. (**A**) Intensity of fluorescence for *S. aureus* ATCC29213, and MRSA strains after AS*yycF* overexpression (scale bar = 100 μm). (**B**) Intensity of fluorescence comparisons and the intensities of *S. aureus* ATCC29213 were measured as reference (*n* = 10, * *p* < 0.05). (**C**) E-test for the sensitivity of MRSA to vancomycin.

**Figure 4:**
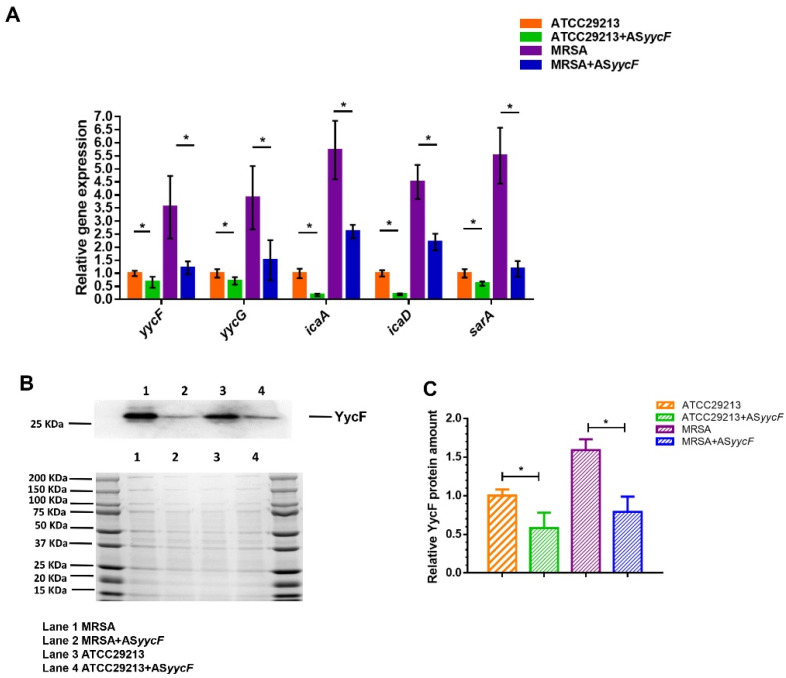
AS*yycF* overexpression inhibited the transcription of biofilm-related genes. (**A**) Quantitative RT-PCR analysis showed the gene transcription in *S. aureus*, using 16S as an internal control (*n* = 10, * *p* < 0.05); (**B**) The productions of YycF were quantified in the cells of *S. aureus* for Western blotting (upper lane); the lower panel shows a Coomassie-stained gel supporting equal loading of each protein sample. (**C**) Quantitative analysis for the relative YycF protein amounts (*n* = 4, * *p* < 0.05).

The AS*yycF*-overexpressing MRSA and ATCC29213 strains also showed lower fluorescence intensity than the MRSA and ATCC29213 strains, at half of their parental strains (Figure 3A,B). Vancomycin is the primary option for methicillin-resistant *Staphylococcus aureus* (MRSA) infections. By E-test, the sensitivity of MRSA to vancomycin decreased from 3 to 1 mg/L after AS*yycF* overexpression (Figure 3C). AS*yycF* downregulated the YycFG pathway and associated virulence gene expression. RT-PCR showed that the expression levels of *icaA*, *icaD*, *sarA*, *yycF*, and *yycG* were significantly decreased in AS*yycF*-overexpressing MRSA and ATCC29213 strains compared with the parental MRSA and ATCC29213 strains (Figure 4A). Western blotting probing with anti-YycF antibody showed that the production of YycF protein was significantly lowest in AS*yycF* ATCC29213 cells among all the groups (Figure 4B,C).

### 2.3. ASyycF Inhibited MRSA Invasion in a Rat Tibial Infection Model

**Figure 5:**
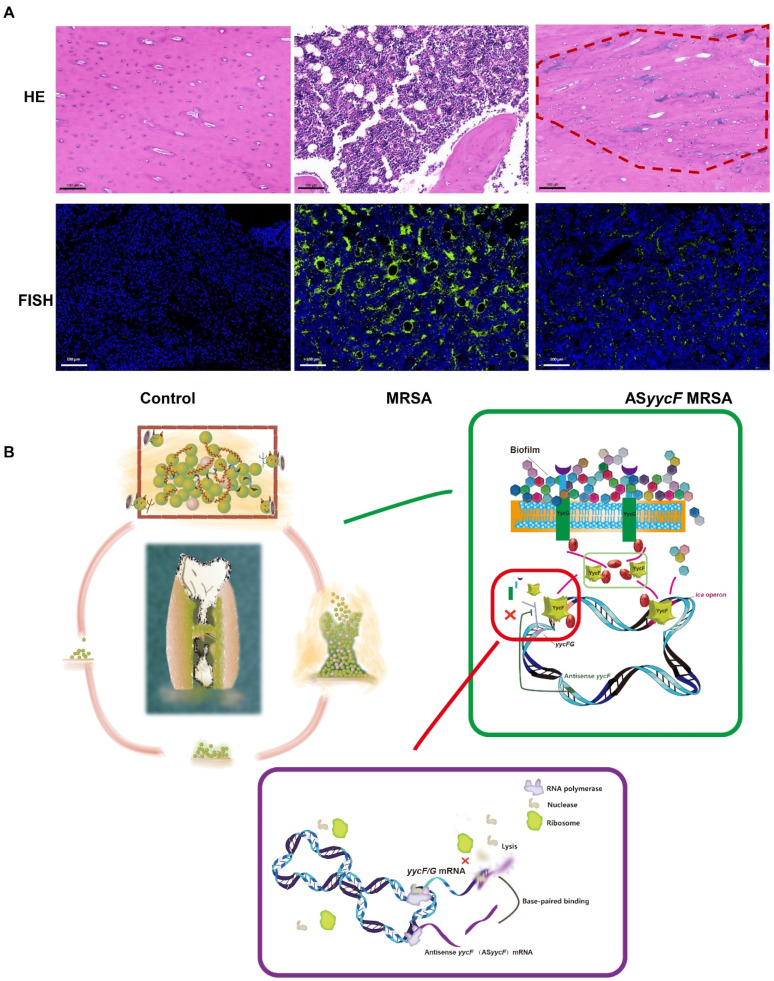
AS*yycF* inhibited MRSA invasion in rat tibial infective model. (**A**) HE staining for histological evaluation (upper lane); the fluorescent labeled peptide nucleic acid in situ hybridization probing for *S. aureus* (lower lane). (**B**) Working model.

MRSA-infected tibia samples at four weeks were collected for histological evaluation, and contralateral uninfected samples were collected as the control group. In the HE-stained samples (Figure 5A, upper lane), substantial destruction in the bone matrix combined with a large amount of inflammatory infiltration was observed in the MRSA group. However, in the AS*yycF* MRSA group, the infective lesions had almost recovered, with signs of bone repair (dashed line area). Using a fluorescently labeled peptide nucleic acid in situ hybridization probe for *S. aureus* 16S rRNA, the *S. aureus* strains were identified as fluorescently labeled green. The fluorescence intensity of the MRSA group was much higher than that of the AS*yycF* MRSA group (Figure 5A, bottom lane).

## 3. Discussion

*S. aureus* is one of the major pathogens of hospital- and community-acquired infections, resulting in a crisis for human health. Additionally, continued antibiotic exposure has enabled it to acquire resistance to most antibiotics, with the result that the population of MRSA accounts for at least 25 to 50% of *S. aureus* infections in hospitals [17]. Compared with methicillin-susceptible strains, MRSA infections have higher mortality rates and health care costs [18]. Biofilm formation is an essential mechanism for the resistance of *S. aureus* to antibiotics and innate host defense. Hence, many investigations have focused on developing novel therapeutic strategies, such as oligonucleotide-based antibacterial strategies to tackle biofilm-associated infections [5,19,20]. In our current study, an endogenous antisense RNA was identified that could be base-paired with *yycF* mRNA (Figure 1).

Once antisense RNA binds to its base-paired mRNA, the expression of the target mRNA and the associated downstream translation process can be modulated [15,16]. For the YycFG TCS, the influence could cause multiple alterations in essential cellular metabolism. In our results, the growth rates, biofilm formation, and bacterial viability were significantly inhibited by overexpressing antisense *yycF* in the AS*yycF* ATCC29213 and AS*yycF* MRSA strains (Figure 2 and Figure 3A,B). Biofilms are constructed with microorganisms embedded in a self-produced extracellular matrix [21]. As shown in the SEM observations, biofilm accumulation was obviously decreased in antisense *yycF*-overexpressing strains, which indicated that antisense *yycF* could negatively regulate biofilm metabolism (Figure 2C). Indeed, biofilms are usually supposed to decrease the sensitivity of biofilm cells to various antimicrobial agents up to 1000-fold when compared with their planktonic forms [10,21,22]. Vancomycin, as the “gold standard” of treatment, has been applied clinically for serious MRSA infections [23]. In this study, we implemented the vancomycin E-test, which indicated that the sensitivity of MRSA was increased with the destruction of biofilms (Figure 3C).

YycFG is the only essential TCS that regulates the *ica* operon, which synthesizes polysaccharide intercellular adhesin (PIA) and *ica*-dependent biofilms [10]. Our results showed that overexpression of AS*yycF* RNA inhibited the transcription/translation of *yycF-* and *ica*-associated genes in AS*yycF* strains, suggesting that AS*yycF* could restrict *ica*-dependent biofilms via YycFG (Figure 4). Additionally, the expression of the global transcriptional regulator staphylococcal accessory regulator operon (*sarA*) in AS*yycF*-overexpressing strains was reduced (Figure 4). The SarA protein is not only involved in the modulation of different virulence-related genes but also impacts *ica*-independent biofilm production in *S. aureus*, which is more frequently isolated from MRSA infections [24]. It is proposed that AS*yycF* has a combined effect in suppressing *S. aureus* biofilm production and infections. However, further investigations on how AS*yycF* inhibited *ica* and *sarA* via YycFG should be considered.

As a major pathogen for most chronic and recurrent microbial infections in humans, *S. aureus* biofilms are involved in a wide range of infections. Some *S. aureus* infections are caused by multidrug-resistant bacteria and even cause high mortality and morbidity rates [21,25]. In addition to antibiotic resistance, biofilms can evade host immune clearance mechanisms and adhere to medical devices such as orthopedic prostheses [24,26]. Therefore, eradication of biofilms will benefit hosts in controlling infection and tissue recovery. Accordingly, our histology evaluations demonstrated that by intervention with AS*yycF*, the MRSA-infected sites in the rat tibia model tended to recover and featured new bone filling with few colonies (Figure 5A). Based on these findings, AS*yycF* has potential as a novel antibacterial agent for infection treatment (Figure 5B).

## 4. Materials and Methods

### 4.1. Bacterial Growth Conditions

The clinically isolated MRSA strain and the methicillin-sensitive ATCC29213 strain provided by the Department of Laboratory Medicine, West China Hospital, were applied [6]. According to our previous protocol, *S. aureus* strains were cultured in TSB medium to midexponential phase (OD_600_ = 0.5). For biofilm formation, five hundred microliters of *S. aureus* suspension (OD_600_ = 0.5) were dropped in 24-well polystyrene culture plates (Nest Biotechnology, Wuxi, Jiangsu, China) and cultured with 14-mm-diameter glass cover slips (Nest Biotechnology, Wuxi, Jiangsu, China) for 24 h for biofilm formation [1].

### 4.2. 5′-Rapid Amplification of cDNA Ends (5′-RACE) Assay for Antisense yycF

Total RNA from midexponential phase ATCC29213 *S. aureus* strains was purified with a MasterPure RNA Purification Kit (Epicentre; Illumina Inc, Madison, WI, USA). Samples of total RNA were used as templates for the First Strand cDNA Synthesis Kit (Thermo Fisher Scientific, Inc., Shanghai, China). According to our previous study [15], the extracted RNA samples (20 μg) were ligated with the 5′-RACE outer adapter using the FirstChoice RLM-RACE Kit (Ambion; Thermo Fisher Scientific, Inc., Shanghai, China). Then, nested PCR was performed for cDNA amplification following previous thermocycling conditions and sequencing by Sangon Biotech Co., Ltd., Shanghai, China) [15,16]. The oligonucleotide primers (SAPCR) for first-strand synthesis PCR and sense strand primers (SAAS) for PCR amplification are listed in Table 1. The 5′-RACE gene-specific outer primer and inner primers for the 5′-RLM-RACE PCR assays are also listed in Table 1.

### 4.3. ASyycF Mutant Construction

According to the sequencing results noted above, an antisense *yycF* (AS*yycF*) sequence was designed and expressed by the shuttle plasmid pDL278. According to a previous study, AS*yycF*-overexpressing MRSA and ATCC29213 mutants were constructed [27]. Briefly, AS*yycF* sequences were ligated into the pDL278 vector at the BamHI and EcoRI restriction sites to synthesize the recombinant plasmid pDL278 AS*yycF*. Then, the overexpressing strains were acquired by transferring the recombinant pDL278 AS*yycF* plasmid into a midexponential phase bacterial suspension with competence-stimulating peptide (CSP) for a 60 min incubation. TSB plates containing 1000 μg/mL spectinomycin and RT-PCR were used for identification.

### 4.4. Detection of Bacterial Growth and Biofilm Assessment

ATCC29213, AS*yycF* ATCC29213, MRSA, and AS*yycF* MRSA strains were cultured in TSB at 37 °C as previously described [28]. Growth curves were measured at OD_600_ every hour. A crystal violet assay was performed to assess the volume of 24 h biofilms according to our previous study [28]. For the epifluorescence test, these biofilms were labeled with SYTO9 (Invitrogen; Thermo Fisher Scientific, Inc., Shanghai, China). The morphology of biofilms was detected by scanning electron microscopy (SEM; Inspect, Hillsboro, OR, USA), and the samples were prepared as described in our previous protocol [28].

### 4.5. Identification of MIC Values

TSA plates were used for determining the MICs. Two hundred microliters of the suspensions involving ATCC29213, AS*yycF* ATCC29213, MRSA, and AS*yycF* MRSA groups was spread onto TSA plates. Then, the E-test strips were placed and incubated for 24 h at 37 °C. The MIC was identified as the value at which the inhibition zone intersected the scale on the E-strip [29].

### 4.6. cDNA Reverse Transcription for RT-PCR Assays

Total RNA was extracted and purified using the MasterPure^TM^ RNA Purification Kit (Epicentre Technologies, Epicentre, Madison, WI, USA) according to the manufacturer’s instructions. A NanoDrop 2000 spectrophotometer (Thermo Scientific, Waltham, MA, USA) was used to detect the purity and concentration of RNAs. Then, the RevertAid First Strand cDNA Synthesis Kit (Thermo Scientific) was applied to reverse transcribe RNA to cDNA with a random hexamer. The primers used for quantitative real-time polymerase chain reaction (qRT-PCR) assays are listed in Table 1. The 16S rRNA gene was set as an internal control. Expression differences were calculated according to the 2^-ΔΔCT^ method as previously described [10].

### 4.7. Protein Extraction and Western Blotting

*S. aureus* planktonic samples were collected and disrupted mechanically by a FASTPREP Beater apparatus (MP Biomedicals, Irvine, CA, USA) with glass beads 0.1 mm in diameter according to our previous instructions [10]. Subsequently, protein samples were collected by centrifugation (12,000× *g* for 2 min at 4 °C) for Western blotting. A purified YycF-specific rabbit polyclonal antibody (HuaAn Biotechnology, Hangzhou, China) was used as a probe following our previous study [10]. A BioRad ChemiDoc^TM^ MP Imaging System (Hercules, CA, USA) was used to detect the density of blot bands.

### 4.8. Osteomyelitis in an In Vivo Rat Model

Animal experiments were approved by our institutional Animal Welfare Committee. Female Sprague–Dawley rats (260–280 g) were used for experiments conducted as previously described [27]. Briefly, the hind leg anteromedial tibia cortex was exposed, and a 0.1-cm-diameter hole was made deep in the medullary cavity. Two groups, namely, MRSA and AS*yycF* MRSA, were evaluated. After four weeks, the rats were sacrificed, and bone specimens were obtained for further evaluations.

### 4.9. Histological Evaluation and Fluorescent In Situ Hybridization Examination

For histological evaluation, samples were prepared as previously described [27]. Briefly, tibia samples were prepared with 10% neutral buffered formalin and 10% EDTA. Five-micrometer sections were processed with HE staining for observation. A FAM-labeled PNA probe (5′-FAM-GAAGCAAGCTTCTCGTCCG-FAM-3′) targeting *S. aureus* 16S rRNA (Servicebio, Wuhan, China) was applied for fluorescent in situ hybridization examination [15].

### 4.10. Statistical Analysis

One-way ANOVA and pairwise multiple comparisons of Tukey’s test were conducted using SPSS software 18.0 (SPSS, Inc., Chicago, IL, USA). Data are presented as the mean ± SD; *p* < 0.05 was considered a statistically significant difference [15].

## 5. Conclusions

In the current study, an antisense RNA base paired with *yycF* mRNA was identified that contributes to the regulation of essential YycFG TCSs. The reactivity of antisense *yycF* RNA is inversely associated with both the transcription of *yycF* mRNA and the production of the YycF protein. Consequently, overexpression of AS*yycF* significantly downregulated biofilm formation and pathogenicity in *S. aureus* while elevating its antibiotic sensitivity, which was mainly dominated by the YycFG pathway via *ica* and *sarA*. Furthermore, AS*yycF*, as a posttranscriptional modulator of *yycF*, reveals potential as a novel strategy for *S. aureus* infections, such as in the treatment of osteomyelitis.

## Figures and Tables

**Table 1:** Sequences of primers in this study.

Primers	Sequence 5′-3′ (Forward/Reverse)	Reference
RT-qPCR		
*icaA*	5′-GATTATGTAATGTGCTTGGA-3′/ 5′-ACTACTGCTGCGTTAATAAT-3′	[6]
*yycF*	5′-TGGCGAAAGAAGACATCA-3′/ 5′-AACCCGTTACAAATCCTG-3′	[6]
*yycG*	5′-CGGGGCGTTCAAAAGACTTT-3′/ 5′-TCTGAACCTTTGAACACACGT-3′	[6]
*sarA*	5′-AGATGGCCCTTCTTCAAATG-3′/ 5′-CCGCAATAATTCTTGTGACG-3′	This study
*icaD*	5′-ATGGTCAAGCCCAGACAGAG-3′/ 5′-CGTGTTTTCAACATTTAATGCAA-3′	[6]
*16S rRNA*	5′-GTAGGTGGCAAGCGTTATCC-3′/ 5′-CGCACATCAGCGTCAACA-3′	[6]
AS *yycF* detection		
First strand synthesis SAPCR	5′-CGTATTATTAGATATCATGTTACCTGGTCG-3′	This study
AS *yycF* detection SAAS	5′-GTTCACGTGTCATTACTTGTCCCATATG-3′	This study
5′RACE		
5′RACE adapter	5′-GCUGAUGGCGAUGAAUGAAC ACUGCGUUUGCUGGCUUUGAUGAAA-3′	First Choice RLM–RACE (RNA ligase-mediated rapid amplification of cDNA ends), Thermo Scientific
5′ RACE outer primer (c)	5′-GCTGATGGCGATGAATGAACACTG-3′	First Choice RLM–RACE, Thermo Scientific
5′ RACE inner primer (d)	5′-CGCGGATCCGAACACTGCGTTTGCTG GCTTTGATG-3′	First Choice RLM–RACE, Thermo Scientific
Gene specific outer primer (a)	5′-GGCGAAGATATTGAATTAACACATCGTG-3′	This study
Gene specific inner primer (b)	5′-CATATGGGACAAGTAATGACACGTGAAC-3′	This study

## Data Availability

Not Applicable.
